# Validation of the Portuguese version of community attitudes toward people with mental illness (CAMI)

**DOI:** 10.1097/j.pbj.0000000000000175

**Published:** 2022-05-18

**Authors:** Inês Lopes, Raquel Simões de Almeida, António Marques, Rosário Curral, Sara de Sousa

**Affiliations:** a Center for Rehabilitation Research (CIR), Psychosocial Rehabilitation Lab, School of Health, Polytechnic of Porto, Porto, Portugal; b Santa Maria Health School; c Department of Clinical Neurosciences and Mental Health, Faculty of Medicine, University of Porto, Porto, Portugal; d Department of Psychiatry and Mental Health, University Hospital Center of São João, Porto, Portugal.

**Keywords:** community attitudes toward people with mental illness, mental illness, stigma

## Abstract

**Abstract:**

Stigma remains a feature that influences the lifestyle of people with mental illness. Negative attitudes, stereotypes, and discrimination are still prevalent in these people's life. Stigma is considered a public health problem that occurs unconsciously in society, categorizing people. Portugal is the seventh-worst country concerning stigma in Mental Health. There have been few improvements in reducing stigma over time, and there is a great need to create investigations and validate instruments that measure stigma in the population.

**Aim:**

This study aims to address the gaps in the level of studies and normative instruments that measure the stigma of the Portuguese population in the face of mental illness. It, therefore, aims to adapt and validate community attitudes toward people with mental illness (CAMI) culturally and examine its psychometric properties.

**Method:**

The 27-item version of CAMI was translated and back-translated into English, which was analyzed and evaluated by a panel of experts. A sociodemographic survey and CAMI were applied in an online format, in which participated 427 adults representing the Portuguese population in general. Finally, the reliability and validity of the instrument were analyzed.

**Results:**

CAMI showed positive values of reliability and validity but not optimal. The confirmatory factor analysis values satisfactory values that indicate good quality of fit: *x*^2^/df*=*3.296; comparative fit index *=* 0.601; goodness of fit index *=* 0.817; and root mean square error of approximation = 0.073, indicates good quality of fit. Cronbach alpha was different for each factor, but it was positive. Spearman coefficient (ρ = –0.343) obtained a negative but consistent value.

**Conclusions:**

This study contributed to the achievement and validation of new measures to assess the stigma of the general population related to people with mental illness. We must continue to analyze this theme, complete the validation of this instrument, and understand the existing levels of stigma, its predominance in society, and the possible creation and implementation of new measures that support literacy in mental illness and anti-stigma.

## Introduction

Stigma remains a feature that influences the lifestyle of people with mental illness. Negative attitudes, stereotypes, and discrimination are still prevalent in these people's life. There is even scientific evidence that public attitudes have not changed in the last 2 decades.^1^ Stigma affects people with mental illness in various ways: reducing their self-esteem, reducing the quality of life, negatively affecting housing, work and financial situation conditions, and creating barriers in the search for treatment because of shame and embarrassment of people with mental illness.^1^ This way, it is known that the person with mental illness faces the symptoms of the disease and the associated limitations and the stigma and social injustice that comes from it.^2^

According to Erving Goffman (1963), stigma occurs when a person is distinguished as discredited^3^ in social contexts, relates to negative attributes, and there is a distinction of the usual pattern (gender, race, and religion). It is considered a public health problem^4^ that occurs unconsciously in society, categorizing people. This categorization includes the placement of labels, association of negative differences and attributes, separation of the terms “us” from “them,” loss of status, and discrimination.^5^ The public stigma reflects the prejudice present in the general population regarding people with mental illness, leading to discrimination.^6^

According to the World Health Organization (WHO, 2001), it is estimated that 1 in 4 people will suffer some mental disorder during their lifetime.^7^ The figures report that around 450 million people are diagnosed with mental illness.^8^ In the World Health Organization European Region, an estimated 110 million people with mental disorders were estimated in 2015.^9^ The 2008–2009 National Epidemiological Study of Mental Health reveals that the prevalence of mental illness in Portugal reaches 22.9% of the population, most frequently aged between 18 and 34 years.^10^ Research also shows that Portugal is the seventh country in the worst position concerning stigma, particularly the community's opinion, where it is assumed to be challenging to talk to people with serious mental illness. This problem is one of the biggest challenges for health,^11^ with a great social injustice to people suffering from issues of this nature.^12^ And although there are treatments available, only a percentage of these people search for professional help due to the stigma that leads to rejection and discrimination,^7^ a global problem. These statistical data estimate that only 15% to 25% of people suffering from mental disorders receive any treatment.^1^

This has been a research topic widely studied in various disciplines, observing exponential growth in the last decade, directed mainly to the study of stigma at the individual level.^13^ There has also been a growing need to talk about public attitudes toward mental illness in the previous years to reduce stigma and discrimination toward mental illness.^14^ Several studies have evaluated community attitudes, investigating what people would do or what they think most people would do, such as when confronted with a neighbor or a co-worker with mental illness.^15^,^16^ Research has also compiled the impact of stigma, assessing its consequences in various aspects,^17^ considering intercultural factors and the type of society.^18^ Some studies say that developing countries have a more significant stigma associated with mental illness, unlike developed countries.^19^ Others note that women tend to be more positive in Western countries and show less social distance to people with mental illness than men.^3^ Research suggests that stigmatizing attitudes have increased in recent decades.^6^ Others report that there has been a decrease in stigma with advanced information since interpersonal contact with people with the disease can develop comprehensive attitudes and change beliefs.^20^ In the global landscape, regarding public attitude, the data suggest few improvements in the decrease in stigma over time.^1^ Some authors argue that stigma can cause more damage to the individual with mental illness than the experience of mental suffering.^21^ Growing stigma measures have accompanied the proliferation of research on stigma in mental illness in the literature. About 400 new instruments developed since 2004. However, many tools have several shortcomings, justified by the lack of consistency in the definition of stigma mechanisms. To understand the stigma, it is necessary to know how to observe and measure it.^13^ As mentioned above, there are currently several assessment tools that analyze the stigma levels in the population. However, they are not validated and accepted for European cultures^7^ and, particularly, for the Portuguese population.^22^ Hence, there is a great need to create investigations and validate scales that measure stigma in the population.^7^

This study, called validation for the Portuguese population of community attitudes toward people with mental illness (CAMI), contributes to the bridge, to some extent, the existing gaps in normative studies and instruments that measure the stigma of the Portuguese population toward mental illness. It intends, therefore, to translate and culturally adapt the CAMI into Portuguese version, to study this bilateral structure in a sample of the Portuguese population in general, and to examine its psychometric properties, in particular reliability, internal consistency, and validity, by calculating Cronbach alpha and confirmatory factor analysis.

### Methodology

The present article is a validation study. A small fraction of the Portuguese population has been analyzed, and its characteristics and results through a questionnaire applied online by social networks. A convenience sample^23^ was used that included 427 participants. The inclusion criteria were: having Portuguese nationality or being resident in Portugal with good mastery of the Portuguese language; female or male; 18 years of age or older; and access to the Internet and social media.

### Ethical considerations

This study met the ethical standards in research with human beings. It was analyzed and approved by the Ethics Committee of the Polytechnic Institute of Porto. All study participants were informed about the objective of the investigation and the nature of data collection. To participate, individuals agreed and marked the free and informed consent form.^24^,^25^,^26^

### Instrument

The CAMI is an instrument for assessing stigma, initially developed by Taylor & Dear in 1981, for measuring the attitudes of the general public toward people with mental illnesses.^14^,^27^ CAMI's greatest strength is exploiting the public about community treatment centers for people with mental illnesses. Since deinstitutionalization is still a novelty in the care of people with mental illnesses, it is crucial to evaluate the general public's attitudes.^13^ The original scale consists of 40 items of attitudes about mental illness,^14^,^27^ and is divided into 4 subscales: authoritarianism; benevolence; social restriction; and community mental health ideology.^28^ The 40 items version of the instrument was considered extensive and, therefore, over time, smaller, and more intuitive versions of CAMI were created,^29^ namely a version with 27 items^30^ and a shorter 1 with 12 items.^31^ In this study, we used the 27 version items because, in addition to applying to any individual, it allows the analysis of the 3 dimensions of stigma: knowledge, attitudes, and behavior of the general public.^30^,^32^ The scale is divided into 3 subscales: attitudes about social exclusion, feelings of benevolence, tolerance, and support for care in community mental health.^30^ It is a questionnaire, composed of 26 statements and an additional item on attitudes related to employment, with answers of agreement, arranged according to the Likert scale from 1 (strongly agree) to 5 (strongly disagree) that translates into a total score and 4 subtotals. The higher the total value obtained in the CAMI, the fewer stigmatizing attitudes of the community.^30^,^32^

### Cultural validation

For the cultural validation of CAMI, was requested permission by the research group described on the website—*Indigo Network* that contains the instrument, as well the rules for the translation. *“Indigo* is a collaboration of fellow researchers in more than 40 countries around the world, committed to developing knowledge about stigma and discrimination related to mental illness, both in its origins and in its eradication. It is coordinated by Mirja Koschorke, Maria Milenova, Nicole Votruba, and Graham Thornicroft at the Global Mental Health Center at the Institute of Psychiatry, Psychology and Neuroscience at King's College London.*”* ^33^ Linguistic validation was subsequently carried out according to the parameters desired by CAMI.^34^ The English language was translated into Portuguese with linguistic adaptation; the translation was revised by a specific group of experts in the field, composed of 3 translators in the mental health and social sciences fields, with experience translating from English to Portuguese. The experts adapted the text according to Portuguese culture, namely specific expressions and words. For retroversion, a professional translator was involved, who did not know the instrument and the original text of the same, to avoid any influence on the translation of words and/or expressions. Subsequently, the group of experts in the area analyzed the latest version and compared it with the original.^24^,^32^,^35^ Was applied a questionnaire in an online format containing sociodemographic questions such as age, gender, marital status, educational qualifications, employment status, a family member with mental illness, degree of kinship, frequency of contact with that person, known with mental illness, type of relationship, and the frequency with which he contacts that person. The CAMI questionnaire was also applied in an online format immediately after completing the sociodemographic questionnaire.

CAMI's fidelity was studied by verifying its internal consistency. The full scale was analyzed by calculating Cronbach alpha to verify its validation and application in scientific research.^24^,^36^

### Data processing

The data collected through the questionnaire was applied, and the statistical study of the data was carried out through 2 databases: the Statistical Package for The Social Sciences version 26.0 for Windows^37^ and the AMOS Software—Structural Equation Modeling version 24.0 for Windows. Statistical analysis involved descriptive statistical measures (absolute and relative frequencies, means, and standard deviations) and inferential statistics. The significance level to reject the null hypothesis was set at (α) ≤0.05. Cronbach alpha internal consistency coefficient was used to verify the validation and application of CAMI fidelity in scientific research, confirmatory factor analysis (CFA), Student *t* test for a sample, Student *t* test for independent samples and one-way Anova. According to the central limit theorem, the normality of distribution was accepted in samples with a dimension greater than 30. The homogeneity of variances was analyzed with Levènés test.

## Results

A convenience sample was used, namely 427 adults with Portuguese nationality or good mastery of the language. The mean age was 38.2years (SD *=* 12.3years), ranging from a minimum of 18 to a maximum of 73years and among which 350 (82%) were female and 77 male (18%). Most participants were single (39.3%), graduated (51.5%), and employed (78.2%). Only 39.8% assume that they have a family member with mental illness, among whom the majority reported being another (23.2%) and the minority being the spouse (0.7), contact this person daily (15.2%). Most participants said that they know someone with mental illness (78.5%), pointing more to known persons (43.8) and less to classmates (1.9%), and contact this person occasionally (36.8%). Table 1 shows the results and percentages of each sociodemographic questionnaire applied to the 427 study participants. A percentage of 39.8% of respondents say that they have family members with mental illness. More than half are unidentified family members, 20% are parents, and 11.2% are siblings. For 15.2%, the frequency of contact with these family members is daily. A percentage of 78.5% of respondents say they know someone with mental illness. Acquaintances (55.8%), friends (24.8%), and neighbors (7.5%) are the most mentioned. About 37% contact these acquaintances occasionally, and 18.5% do so daily.

**Table 1:** Sociodemographic characterization of sample N** **=** **427

	N	%
Gender		
Female	350	82.0
Male	77	18.0
Age (M; SD)	38.2	12.3
Marital status		
Married	158	37.0
Divorced	32	7.5
Single	168	39.3
Non-marital partnership	62	14.5
Widower	7	1.6
Education degree		
12°Year	62	14.5
4Class	6	1.4
6Class	15	3.5
9°Year	29	6.8
Degree	220	51.5
Other	95	22.2
Employment situation		
Sick leave	8	1.9
Unemployed	34	8.0
Employed	334	78.2
Student	40	9.4
Social pension	6	1.4
Retired	5	1.2

N = number of participants, M = mean, SD = standard deviation, % = percentage.

**Figure 1:**
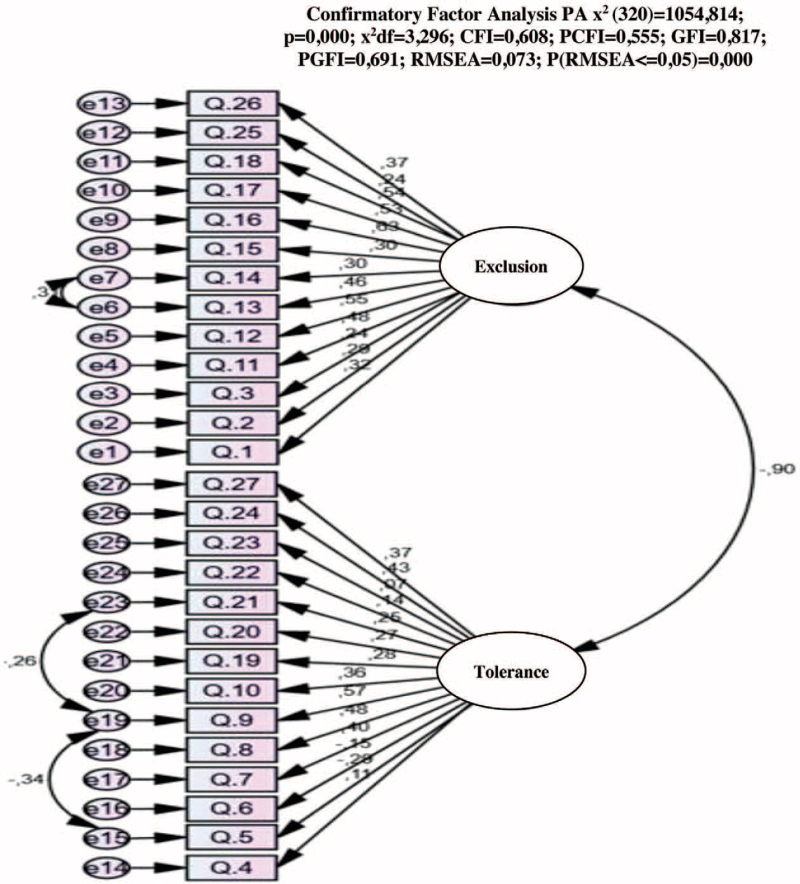
CAMI's confirmatory factor analysis.

The instrument's validity was calculated by analyzing the structure of the CAMI bi-factorial model. All 27 items of the instrument were used, similar to the study conducted by Rüsch et al (2011). This was accomplished through CFA.^30^ The values obtained, *x*^2^/df=3296; comparative fit index = 0.601; goodness of fit index = 0.817; root mean square error of approximation = 0.073, indicate a good quality of adjustment. The adjustment of the model implied the correlation of errors 6 and 7, 15 and 19, and 19 and 23. The values are relatively like those obtained by the authors Rüsch et al (2011), namely *x*2/df=2.242 and RMSEA = 0.059. Figure 1 shows the data obtained through the confirmatory analysis.

The internal consistency, evaluated with Cronbach alpha coefficient, of the prejudices and social exclusion factor was 0.*699*, and that of the tolerance and support in the community factor was 0.634 (Table 2). The descriptive statistics of the variables used in the present study can be evaluated in Table 3. Spearman coefficient obtained a negative correlation, like Rüsch et al,^30^ but slightly lower (ρ = –0.343).

**Table 2:** Internal consistency by Cronbach alpha coefficient

	Cronbach alpha	Number of items
Prejudices and exclusion	0.699	13
Tolerance and support in the community	0.634	14

**Table 3:** Descriptive statistics of the 2 factors: prejudices and social exclusion and tolerance and support in the community

	Minimum	Maximum	Mean	Standard deviation
Prejudices and exclusion	2.62	5.00	4.38	0.41
Tolerance and support in the community	1.07	2.86	1.93	0.32

The value obtained in the prejudices and exclusion factor is significantly higher than the midpoint of the scale, *t* (427) = 69,345, *P* *=* .001. In comparison, the value obtained in the tolerance and support in the community factor is significantly lower than the midpoint of the scale, *t* (427) = –67,798, *P* = .001, which means that there are high values of prejudice and exclusion and low values of tolerance and support in the community for mental illness.

Women have significantly higher values of prejudices and exclusion stemming from mental illness (4.43 vs 4.16), *t* (427) = *5,397*, *P* = .001. The difference in tolerance and support is not statistically significant. The difference in the values obtained in the CAMI between subjects with relatives with mental illness and individuals who do not have relatives with mental illness is not statistically significant. People who know someone with mental illness have significantly higher values of prejudices and exclusion stemming from mental illness (4.40 vs 4.28), *t* (425) = 2,450, *P* *=* .015. Subjects who know someone with mental illness have a significantly lower tolerance and support for mental illness (1.91 vs 2.01), *t* (425) = 2,661, *P* = .008. Subjects over 45years of age have significantly lower values of prejudices and exclusion stemming from mental illness, *F* (2.424) = 14,497, *P* = .001. The differences in tolerance and support factor according to age are not statistically significant.

## Discussion

Based on a representative survey of the Portuguese adult population, the present study provides evidence about stigmatizing attitudes toward people with mental illness through applying the stigma assessment instrument, CAMI, from which we intend to develop the Portuguese version. Since its development, CAMI has been considered internationally as a standard instrument for assessing the stigma of the population vis-à-vis people with mental illness. Its excellent qualities are that it does not use vignettes, which allows a reasonable margin of representation of the scale, and the fact that it is quite intuitive, reflecting precisely the concept of stigma in its dimensions.^32^ It is also considered a measure with positive psychometric information regarding its reliability, validity, and dimensionality.^13^ This scale is validated in several languages such as Finnish, Lithuanian, Italian, Swedish, Portuguese, Greek, and Thai. In general, existing studies show a positive consistency in which Cronbach alpha ranges from 0.6 to 0.9.^13^,^29^,^32^ For these reasons, it has served as an engine for carrying out several anti-stigma campaigns.^14^,^32^ Instrument validations have been made for several countries and populations. However, few stigma assessments instruments for the Portuguese population, namely the CAMI.^22^ Given the above, it became essential to realize whether by adapting the CAMI version of 27 items to the Portuguese language, its psychometric properties remained. Thus, this study aimed to examine the psychometric characteristics of the CAMI, using a sample of 427 individuals to assess the reliability and validity of the bi factorial structure.

The translation, cultural, and linguistic adaptation into Portuguese followed the guidelines established internationally and by *Indigo Network,* from which the instrument was removed to ensure the quality of the translation. This study allowed consolidating the cultural adaptation of CAMI carried out by Rüsch et al^30^ since there were no significant changes during the process.

Regarding the sample of this study, according to the research carried out so far, this was the first sample created in Portugal, specifically to study the psychometric properties of CAMI for the general population.^22^

In Portugal, within the sample studied, there were high values of prejudice and exclusion and low values of tolerance and support in the community. Compared to the study by Rüsch et al^30^ or the study by Högberg et al,^38^ or Deverick et al,^21^ these values are contradictory. These results confirm that some attitudes toward people with mental illness are associated with sociodemographic characteristics such as age, gender, and contact with mentally ill people as in some other studies.^14^,^39^,^40^

The younger people have higher levels of exclusion and tolerance, although the latter value is statistically insignificant. These values align with some studies that state that older people have more positive attitudes toward mental illness.^14^,^22^ In the same way that the incidence of the female gender was higher, women also showed higher levels of prejudice and exclusion than of tolerance, unlike studies which refer to being the gender with more positive attitudes toward mental illness.^14^,^22^ As for contact with family members with mental illness, it is impossible to take many relations since the results were statistically insignificant. However, in contact with acquaintances with mental illness, there were high levels of prejudice and low levels of tolerance. Some studies show that people who contact the mentally ill show higher levels of stigma, whether known or family members, resulting in negative consequences for these people.^22^,^39^

The Cronbach alpha value, which verifies the internal consistency of the 2 CAMI factors, reveals that factor 1 corresponding to social exclusion is more consistent. However, both factors have an alpha above 0.6; that is, the analysis of the results allows us to state that the Portuguese version of the CAMI presents a positive but not optimal internal consistency.^41^ Concerning CAMI validation studies in other countries such as Ochoa et al 20 1 6,^29^ or Morris et al 20 1 2,^42^ or Högberg et al 2012,^38^ Cronbach alpha value has more positive values. When comparing these results with the pilot study results, it was found that most are within the reference values. Thus, the value of internal consistency does not meet what is desired, even though it approaches what is usually considered an acceptable value.

Using CFA procedures, the instrument validity results provided values that indicate good quality of the adjustment with acceptable correlation values, which means that the bi factorial structure used is adequate to the data obtained^43^,^44^ similarly to the study by Rüsch et al 2011.^30^ Other CAMI validation studies from other countries and contexts used different factor models, namely the research by Wolff et al,^40^ who obtained very satisfactory results but proceeded to the CFA with division by 3 factors, as well as Tong et al 2020.^45^ Goh et al 2021^28^ divided the CAMI into 4 elements and found that it was not viable until it tested the division into 3 factors and obtained favorable results. The research by Garcia et al 2017^32^ suggested a 4-factor analysis and a 1-factor analysis, the first of which yielded optimal values. A recent study by Morris et al 2012^42^ tested de division by several factors found that the most acceptable results are verified when the CAMI is divided into 3 aspects.

The criterion validity, analyzed using Spearman correlation coefficient, suggests that the obtained value is slightly undersized. In the study by Rüsch et al, the correlation was also negative, but with a more significant value. However, both instruments are negatively correlated, conferring some rigor to the study.^46^ In other CAMI validation surveys, such as Garcia et al^32^ and Fox et al,^13^ although with different factor divisions, the criterion validity also presented favorable values and positive information. It is noteworthy that the value of the correlation can be influenced by the number of items in the instrument and, therefore, some studies that change the original instrument, as is the case, for example, in the research by Garcia et al^32^ and Sun et al 2014.^47^ CAMI validation studies tend to have higher correlation values in the more extended versions as in the studies by Ochoa et al 2016^29^ and Sun et al 2014.^47^

It is important to consider that there are always limitations arising from random factors that can influence the results of studies. The main limitations of this study were the crosssectional approach and the bias of the sample selection and outcomes. The sample number was reduced, and the participant's characteristics are not precisely known, raising doubts about the accuracy of the answers. Since the surveys were applied through an online form, it may have called into question the coherence at a distance. As conditions in filling out the questionnaire, particularly the discrepancy of responses by gender and education level, this decision was taken due to COVID 19. The second major limitation was that the present study was based on the pilot study by Rüsch et al (2011) which may have conditioned some results, namely the value of internal consistency since the methodology was completely oriented. And the validation studies referred to use different types of populations, making it challenging to compare the results.

## Conclusions

Although there are scales developed worldwide to assess stigma in the general population, it was decided to investigate the validity of CAMI for the Portuguese population because it is easy and quick to apply and simple to understand. CAMI validation was the main focus of the present study. Still, it was felt that it was also essential to study the characteristics of the participants and, subsequently, correlate the information obtained with the application of the CAMI instrument. Several studies in different countries and varying types of population have obtained similar and/or different results with several modifications of CAMI, raising doubts about the instrument's real validity. However, the results of this study reveal that the 27-item version of the CAMI in Portuguese has positive levels of reliability and validity, has maintained most of the psychometric characteristics of the pilot study. Thus, it can be affirmed that this study contributed to obtaining and validating new measures to assess the stigma of the general population to people with mental illness. The survey also reveals that many participants have many negative attitudes toward people with mental illness. These results can have adverse consequences for the care and recovery of these people. There is a great need to create more research that will provide us with information about the most effective methods of assistance and treatment of people with mental illness in the Portuguese community. In short, this work is not a final product but a contribution to realizing others that will benefit the community. The further analysis of this theme—CAMI validation, is fundamental to understand, over time, the existing levels of stigma, its predominance in society and the possible creation and implementation of new measures that support literacy in mental illness and anti-stigma.

## Conflicts of interest

The authors report no conflicts of interest.
